# News and Items

**Published:** 1868-10-15

**Authors:** 


### *News and Items.*

*Obituary.*—Dr. Joseph N. McDowell, one of the most distinguished surgeons in the West, and a resident of St. Louis for twenty-eight years, died September 25th, of congestive chills, in the sixty-third year of his age. Professor McDowell will long be remembered for great professional sagacity and tact, and especially for eccentricities of genius which have made his name famous.

*Christian Friedrich Shoenbein*, the eminent chemist, died at Baden Baden about the 1st of September. He will be especially recollected as the discoverer of ozone; also of antozone, and the inventor of gun cotton.

*President Haven* strongly advises the admission, as students, into the Michigan University, of women, on the same condition as men. He likewise contends that, in the Medical Department, the Professors should be chosen for their scientific acquirements, irrespective of whether they are allopathic or homœopathic.—*Chicago Tribune Telegram.*

Whether this involves admission of females to the Medical Department, we are not informed.

*Dr. J. Marion Sims* has been elected a corresponding member of the Obstetrical Society of Berlin. He is well worthy of such honor.

*Prof. L. C. Lane*, of San Francisco, was recently made the victim of an annoying suit for malpractice. The prosecutor took more than a year of time to get up his case, and yet, when the evidence was concluded in court, the case was so clearly a bare-faced attempt to extort money, that the judge refused to permit it to go to the jury.

The mortality for July last, in San Francisco, was 346. This is said to be unprecedented in the history of the city. For the three summer months, the thermometer did not rise above 75 ° at noon-day, and was not as high as 70 ° for more than ten days. During the same period there were not more than twenty days of clear sky, from sunrise to sunset. Yet one-third of the cases of death were from disorders of the bowels, a class of diseases usually ascribed to the heat of summer.

A child died recently in Jackson, Ohio, from the effects of the oil from a tobacco pipe, applied to a burn on the lip. The little one was soon after seized with convulsions, and died within twenty-four hours.

*Dr. M. H. Houston* records, in the Richmond and Louisville *Medical Journal*, a case of abnormal gestation, where, four years after conception, the fœtal bones were discharged through the rectum. The patient, at last accounts, was in fair prospect of recovery. Quite a number of queer cases of monstrosities are chronicled by the medical press as of recent occurrence. Philosophical considerations of identity are puzzling observers. Individuals and sexes are mixed in most glorious confusion.

*Captain John Travis*, of pistol fame, is now in this city. It is difficult to conceive of an instance showing more remarkable cultivation of steadiness of muscle. Many will recollect the great feat he performed in Louisiana in 1853, shooting, at the first shot, on a wager of one thousand dollars, an orange off the head of John P. Osgood, at the distance of twenty paces. Another great feat of his, at Niagara Falls, in 1857, was much talked of at the time. Monsieur Blondin, the renowned rope walker, was crossing the Niagara river from the American to the Canada side; when half way across, Blondin held out his hat, and the Captain, from the deck of the steamer "Maid of the Mist," at a distance of three hundred and sixty feet, shot a ball through the hat. Recently he shot seven birds out of ten, with his pistol, "ten yards rise and ten yards boundary." Shot No. 35 to the pound. The day, unfortunately, was windy, or he would undoubtedly have killed all the ten birds. The feat may well be said to be a remarkable one, and "deserves to be classed among the most marvelous trials of skill." Physiologically, there seems no limit to the degree of development of either human mind or muscle.

*Francis H. Ramsbotham*, the eminent physician, and author of a well known work on Obstetric Medicine and Surgery, died on the 6th of July last, in his sixty-eighth year.

A Paris correspondent of the *Medical Record* gives the details of a case where an insane woman, sixty-four years old, swallowed a silver fork for the purpose of committing suicide. After ten months sojourn and peregrinations in the stomach and intestines, it was ultimately discharged through a fistulous opening to the left of the umbilicus. The patient recovered. [This case was first reported in the *N. Y. Med. Journal*, as we learn since the above was in type, some eighteen months since.]

*Removing Tan.*—Tan may be removed from the face by mixing magnesia in soft water to the consistency of paste, which should then be spread on the face and allowed to remain a minute or two. Then wash off with Castile soap-suds, and rinse with soft water.—*Chemical News.*

*Oil Stains in Marble.*—These can be removed by applying common clay, saturated with benzole. If the grease has remained long enough, it will have become acidulated, and may injure the polish, but the stains will be removed.—*Chem. and Drug.*

*Chlorodyne.* — Ed. McIngall, Jr. (St. Louis *Medical Reporter*), gives the following formula as the best method of preparing the compound:

| | |
|---|---|
| Take of sulphate of morphia, | gr. lxiv. |
| Alcohol, 95 per cent., | f ℥ ij. |
| Chloroform, purif., | f ℥ vj. |
| Sulphuric acid, | q. s. |
| Ext. cannabis ind. (Allen's) | ʒ ss. |
| Oleoresin of capsicum, | gtt. xij. |
| Hydrocyanic acid (Scheele's) | gtt. xcvj. |

Shake together the sulphate of morphia, alcohol and chloroform, then add the sulphuric acid, shake well until it becomes clear, then add the oleoresin of capsicum, ext. cannabis and hydrocyanic acid.

---

*Dr. Leland A. Babcock's Silver Uterine Supporter.*

BALTIMORE, August 14, 1

L. A. BABCOCK, M.D., *Freeport, Ill.*

MY DEAR SIR:—I find your Uterine Supporter the desideratum in displacements of that organ. All former appliances for this purpose must ere long be classed with the things of the past.

Its advantages are so apparent that no intelligent physician will use any other supporter, after observing its action in a single case of prolapsus uteri.

I shall take pleasure in recommending it to my patients, and presenting it to my classes in Washington University.

I am dear sir, very respectfully, your obedient servant,

HARVEY L. BYRD, M.D.,
*Professor of Obstetrics in the Medical Department of Washington University, of Baltimore, Maryland.*

